# Drug Utilization and Safety Profile of Intravenous Iron Isomaltoside 1000 in Cancer Patients With Iron Deficiency Anemia: A Retrospective Study

**DOI:** 10.7759/cureus.106488

**Published:** 2026-04-05

**Authors:** Kartik Kumar Anil Kumar Purohit, Mitesh Kumar Dilip Kumar Halvawala

**Affiliations:** 1 Hematology and Oncology, Zydus Hospital, Vadodara, IND; 2 Hematology, Sanguine Clinic, Surat, IND

**Keywords:** cancer-associated anemia, drug utilization, intravenous iron, iron deficiency anemia, iron isomaltoside 1000

## Abstract

Background: Iron deficiency anemia (IDA) is a persistent complication in cancer patients and may result from chronic inflammation, poor nutritional intake, and myelosuppressive therapies. Intravenous (IV) iron preparations, such as iron isomaltoside 1000, allow rapid iron repletion. This study evaluated the drug utilization patterns and safety profile of IV iron isomaltoside 1000 in cancer patients with IDA in a real-world setting.

Methods: This retrospective observational study included 100 adult cancer patients diagnosed with IDA who received IV iron isomaltoside 1000 at two participating centers between January 2024 and September 2025. Demographic data, laboratory parameters (hemoglobin, serum ferritin, total iron-binding capacity (TIBC), and transferrin saturation (TSAT)), dosing details, and adverse events (AEs) were recorded. Continuous variables were expressed as mean ± standard deviation (SD), and paired t-tests were used to compare pre- and post-treatment laboratory values. A p-value less than 0.05 was considered statistically significant.

Results: The mean age of patients was 49.5 ± 17.9 years, and 51% were female individuals. The mean baseline hemoglobin was 8.8 ± 1.9 g/dL and improved to 11.68 g/dL (p < 0.001) after one month of treatment. Among the 92 patients with paired baseline and one-month follow-up data, serum ferritin increased from 10.24 ng/mL to 133.16 ng/mL and TSAT improved from 13.28% to 21.98% (p < 0.001), while TIBC decreased from 441.62 µg/dL to 278.00 µg/dL (p < 0.001). AEs occurred in 7% of patients, including Fishbane reaction in 6% and mild musculoskeletal pain in 1%; all were mild and self-limiting.

Conclusion: IV iron isomaltoside 1000 was associated with significant improvement in hematologic parameters and demonstrated a favorable safety profile in cancer patients with IDA. It can be considered a safe and practical option for rapid iron repletion in cancer management.

## Introduction

Cancer-associated anemia (CAA) is a prevalent and clinically significant complication observed in patients with malignancies, affecting a substantial proportion both at diagnosis and during the course of treatment [1]. Worldwide, anemia has been reported in 30% to 90% of patients with cancer, depending on tumor type, stage, treatment status, and the definition of anemia used [1]. CAA has a reported prevalence of 46.5% to 61% in India [2]. Anemia in cancer patients is multifactorial and may occur as a direct consequence of the malignancy, as an adverse effect of anticancer treatments, or due to tumor-derived cytokines and other bioactive substances that interfere with erythropoiesis. The condition cannot be completely explained by the traditionally described causes such as bone marrow infiltration, blood loss, hemolysis, renal, hepatic or endocrine disorders, or nutritional deficiencies. Cancer-related anemia is now thought to be caused by a complex interaction between the tumor cells and the host immune system, which ultimately disrupts normal erythropoiesis [3].

CAA contributes to tumor hypoxia, which in turn promotes angiogenesis and diminishes the effectiveness of chemotherapy and radiotherapy [3]. CAA is associated with an overall adverse prognosis, particularly in patients with lung, prostate, head and neck cancers, and lymphoma, where it leads to a significant reduction in survival compared to their non-anemic counterparts [4]. Additionally, CAA results in fatigue, impaired quality of life (QoL), and reduced physical activity, while also contributing to lower treatment response rates, decreased disease-free survival, and reduced overall survival (OS) [5]. Therefore, timely and effective correction of CAA is critical in comprehensive cancer care.

The current treatment modalities for CAA include red blood cell transfusions, erythropoiesis-stimulating agents (ESAs), and iron supplementation. Transfusions provide rapid correction of severe anemia but carry risks such as transfusion reactions, and repeated transfusions may lead to iron overload; therefore, they are generally reserved for urgent or refractory cases [6]. ESAs stimulate red blood cell production and can reduce the need for transfusions; however, their use is limited by concerns over thromboembolic events and potential tumor progression, restricting their indication primarily to patients undergoing chemotherapy with palliative intent. Iron supplementation, particularly intravenous (IV) iron, plays a crucial role in managing both absolute and functional iron deficiency, which is common in cancer patients due to inflammation-induced iron sequestration. Among IV iron formulations, ferric carboxymaltose and iron isomaltoside 1000 are commonly used options due to their ability to deliver larger doses per infusion and rapidly replenish iron stores [4].

Current guidance for CAA emphasizes assessment of iron status and supports the use of IV iron in selected patients with iron deficiency as part of supportive anemia management [7]. Despite the availability of various treatment options for CAA, challenges remain in optimizing anemia management, particularly in the context of inflammation-driven functional iron deficiency and the need for safe, effective, and convenient iron repletion therapies [8,9]. Limited data exist on the efficacy and safety of newer IV iron formulations, such as iron isomaltoside, specifically in Indian cancer patients who often present with advanced disease and multiple comorbidities. This study aimed to address this gap by evaluating the real-world utilization of iron isomaltoside 1000 (Feruno®) in cancer patients diagnosed with iron deficiency anemia (IDA), specifically examining calculated iron requirements and actual doses administered, as well as the drug's safety profile.

## Materials and methods

Study design

This was a retrospective, observational, multicenter study conducted to evaluate the drug utilization patterns and safety profile of IV iron isomaltoside 1000 (Feruno®) in adult cancer patients diagnosed with IDA. Data were retrospectively collected from patient case files and electronic medical records of 100 cancer patients treated with iron isomaltoside 1000 for IDA as part of standard clinical practice at Zydus Hospital, Vadodara, Gujarat, India, and Sanguine Clinic, Surat, Gujarat, India. Cancer patients diagnosed with IDA on the basis of iron studies were included. Absolute iron deficiency was defined as serum ferritin <30 ng/mL, and functional iron deficiency was defined as serum ferritin 30-100 ng/mL with transferrin saturation (TSAT) <20%. Patients meeting either of these criteria and prescribed iron isomaltoside 1000 during the study period were included. Patients whose anemia was caused by factors other than iron deficiency were excluded from the analysis.

Study objectives

The primary objective of this study was to evaluate the utilization pattern of IV iron isomaltoside 1000 in cancer patients with IDA, including assessment of the estimated iron requirement and the actual dose administered in routine clinical practice. The secondary objective was to assess the safety profile of iron isomaltoside 1000 by documenting the incidence, type, and severity of adverse events (AEs) observed during the treatment period.

Study population and sample size calculation

The study included all eligible cancer patients with IDA who received IV iron isomaltoside 1000 during the study period (January 2024 to September 2025). All eligible patients meeting the inclusion criteria during the study period were consecutively included. No separate sampling method or sample size calculation was applied because this was a retrospective study. Missing data were handled using available-case analysis; patients with missing paired follow-up values were excluded only from the corresponding comparative analyses. A total of 100 patient records meeting the inclusion criteria were available and included in the analysis.

Data collection and outcome measures

Relevant demographic and clinical information were extracted from patient case files and electronic medical records, including age, sex, weight, baseline hemoglobin levels, total iron-binding capacity (TIBC), serum ferritin, and TSAT. Details regarding iron dosing (total dose administered), dosing schedule, and timing of follow-up were recorded from January 2024 to September 2025. Iron requirement was estimated using the simplified weight- and hemoglobin-based dosing approach followed in routine clinical practice at the study centers, and the actual administered dose was recorded from the medical records. Drug utilization patterns were then evaluated by comparing the estimated iron requirement with the dose actually administered across clinical subgroups defined by gender, baseline hemoglobin category, and body weight. Sankey diagrams were used as descriptive visualization tools to illustrate the distribution of patients across these variables and the administered iron dose, using Tableau (Salesforce, Inc., San Francisco, USA). The primary outcome measure was to analyze the pattern of iron isomaltoside 1000 utilization, including variability in dosing practices across demographic and clinical subgroups. The secondary outcome was to assess the safety of Feruno® by identifying the incidence and type of AEs documented during or after administration. AEs were categorized and analyzed for frequency, with emphasis on infusion-related reactions like Fishbane reaction and mild musculoskeletal pain. Iron requirement was estimated using a simplified weight- and hemoglobin-based dosing approach applied in routine clinical practice, and the administered dose was then recorded from the medical record.

Changes in clinical parameters such as hemoglobin, ferritin, and TSAT following Feruno® administration were also recorded, where available, to support evaluation of treatment response.

Statistical analysis

This study utilized descriptive statistical methods to analyze the data. Continuous variables were summarized using mean and standard deviation (SD). Categorical variables, including gender distribution, hemoglobin categories, and incidence of AEs, were presented as counts and percentages. Comparative analyses of clinical parameters before and after iron isomaltoside administration were performed using paired t-tests on matched observations. The paired t-test was selected because analyses were based on within-subject pre- and post-treatment differences, and the distribution of these differences was assessed for approximate normality before parametric testing. For comparative outcomes, test statistics, degrees of freedom, p-values, and 95% confidence intervals for mean differences were reported. A p-value < 0.05 was considered statistically significant.

Ethical considerations

The study was conducted in accordance with the ethical principles outlined in the Declaration of Helsinki. This was a retrospective, non-interventional, multicenter chart review utilizing fully anonymized/de-identified patient records with no direct patient contact or alteration of standard clinical care. The study was conducted in accordance with institutional policies applicable to retrospective record-review studies. As only de-identified data were analyzed and no intervention was performed, the requirement for individual informed consent was waived. No separate ethics committee name or approval/waiver reference number was available in the study records.

## Results

Baseline demographics

The mean age of the study population was 49.5 ± 17.9 years, and the mean body weight was 58.6 ± 9.4 kg. Baseline laboratory parameters revealed a mean hemoglobin level of 8.8 ± 1.9 g/dL. The hemoglobin categories used for the utilization analysis are presented in Table 1.

**Table 1:** Baseline demographic and clinical characteristics of the study population (N = 100) Hb: hemoglobin; TIBC: total iron binding capacity; TSAT: transferrin saturation

Parameter	Value
Age (years) (mean ± SD)	49.5 ± 17.9
Weight (kg) (mean ± SD)	58.6 ± 9.4
Gender	
Female, n (%)	51 (51%)
Male, n (%)	49 (49%)
Hb (g/dL) (mean ± SD)	8.8 ± 1.9
<10 g/dL, n (%)	21 (21%)
≥10 g/dL, n (%)	79 (79%)
TIBC (µg/dL) (mean)	441.6 ± 80.32
Serum ferritin (ng/mL) (mean)	58.2 ± 176.4
TSAT (%) (mean)	11.7 ± 7.6

Drug utilization patterns

**Figure 1:**
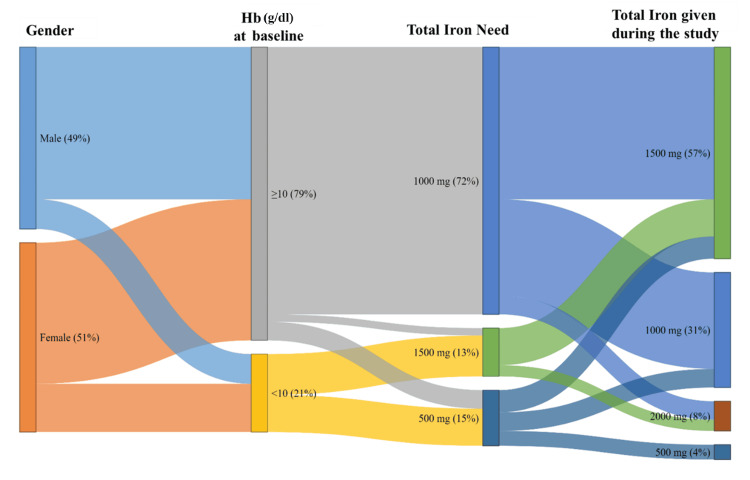
Sankey diagram showing the distribution of patients by gender, baseline hemoglobin (Hb) category, estimated iron requirement, and administered iron dose (N = 100) The diagram was created using Tableau (Salesforce, Inc., San Francisco, USA).

All 100 patients received IV iron isomaltoside 1000 as per clinical assessment of their iron requirements. Dosing was individualized based on baseline hemoglobin levels, gender, and body weight. The majority of patients received their total calculated dose in a single or split infusion during the initial treatment visit. The Sankey diagram descriptively illustrates the distribution of administered iron doses according to gender, baseline hemoglobin category, and estimated iron requirement (Figure 1).

Change in clinical parameters

**Figure 2:**
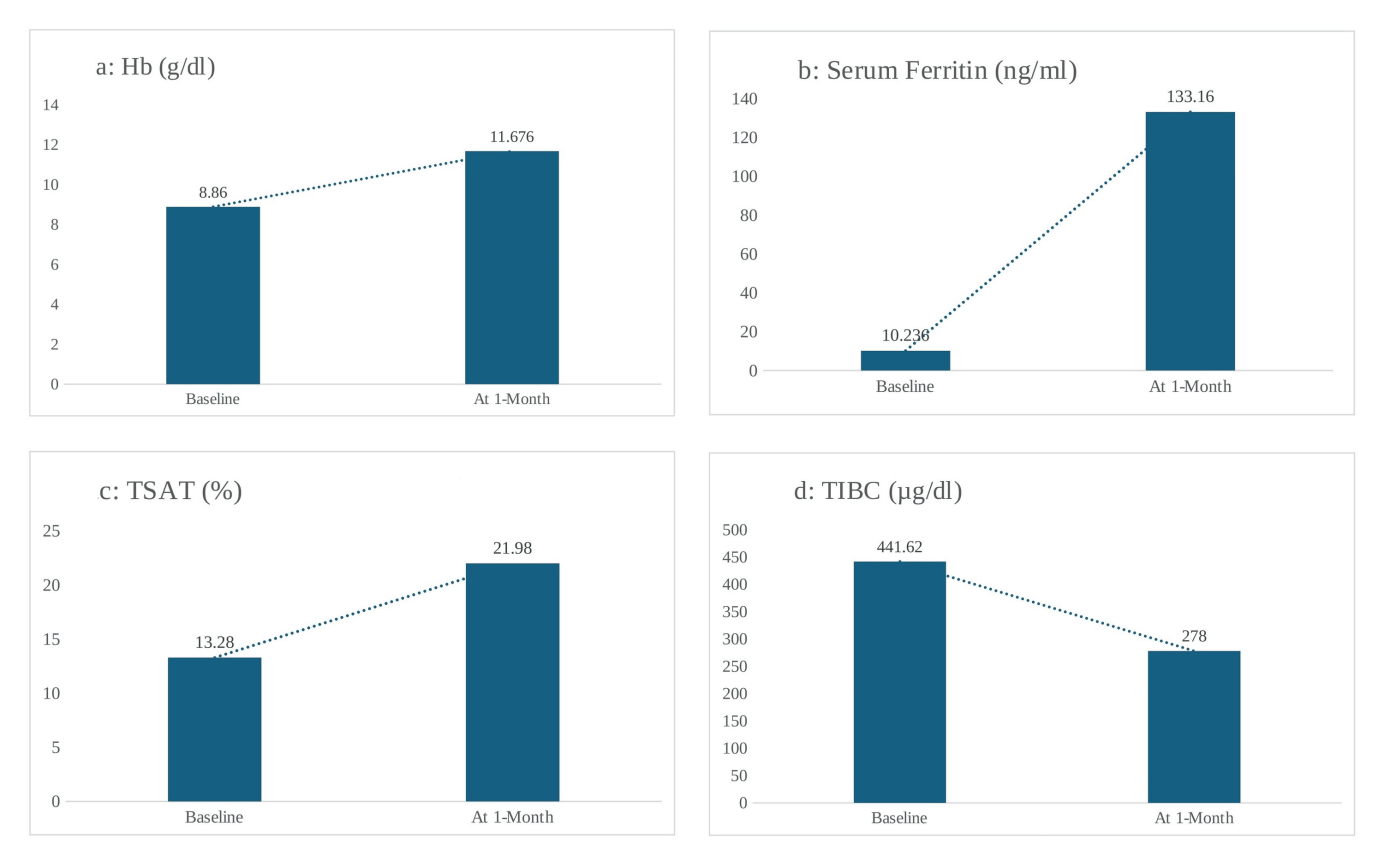
Comparison of clinical parameters before and one month after treatment with intravenous iron isomaltoside 1000 (N = 92): (a) hemoglobin (Hb), (b) serum ferritin, (c) transferrin saturation (TSAT), and (d) total iron-binding capacity (TIBC) Values were compared using paired t-tests (df = 91), and a p-value < 0.05 was considered statistically significant.

Among the 100 patients included in the study, paired baseline and one-month follow-up laboratory data were available for 92 patients; the remaining eight patients lacked complete paired follow-up laboratory values in the medical record and were therefore excluded from the paired comparative analysis (paired t-test, df = 91). In the full study cohort (N = 100), baseline serum ferritin was 58.2 ± 176.4 ng/mL and baseline TSAT was 11.7 ± 7.6%, as shown in Table 1. Among the 92 patients included in the paired analysis, mean serum ferritin increased from 10.24 ± 6.09 ng/mL at baseline to 133.16 ± 105.23 ng/mL at one month (t = 8.25, p < 0.001); mean hemoglobin increased from 8.86 ± 1.90 g/dL to 11.68 ± 1.46 g/dL (t = 20.84, p < 0.001); and mean TSAT increased from 13.28 ± 5.25% at baseline to 21.98 ± 5.10% at one month (t = 18.49, p < 0.001). In contrast, TIBC decreased from 441.62 ± 80.32 µg/dL to 278.00 ± 76.92 µg/dL (t = -17.01, p < 0.001) (Figure 2).

Safety profile

Out of 100 patients treated with iron isomaltoside 1000, AEs were reported in seven patients (7%), while 93% reported no treatment-related complaints. The most common AE was Fishbane reaction in six patients (6%), presenting as transient flushing, myalgia, or chest tightness during infusion. One patient (1%) reported mild musculoskeletal pain following administration. All reported AEs were mild, self-limiting, and did not require discontinuation of therapy. No serious adverse events (SAEs) were observed. Because this was a retrospective chart review, formal severity grading, precise time of onset, and detailed management measures were not uniformly documented in all records.

## Discussion

This retrospective study evaluated the drug utilization patterns and safety profile of iron isomaltoside 1000 (Feruno®) in 100 cancer patients with IDA. The analysis revealed appropriate clinical alignment in dosing practices based on hemoglobin levels, gender, and weight. A single or split infusion approach was commonly adopted, and treatment was well tolerated. Post-treatment evaluations showed significant improvements in hemoglobin, serum ferritin, and TSAT, and a reduction in TIBC levels. The incidence of AEs was low and predominantly mild, with no serious or life-threatening reactions reported. These findings suggest that iron isomaltoside 1000 is both effective and safe in correcting IDA among oncology patients in real-world settings.

The primary outcome of this study focused on utilization trends. Most patients received a single, high-dose infusion or appropriately split dosing based on calculated iron requirements. This aligns with findings by Kalra et al. (2016) in patients with chronic kidney disease (CKD), where higher initial dosing (>1000 mg) led to better hemoglobin response and lower retreatment rates [10]. The descriptive utilization analysis indicated that dosing varied according to baseline hemoglobin, gender, and weight. Such optimized utilization is especially important in oncology, where reducing infusion frequency may simplify treatment logistics and potentially reduce healthcare burden.

The improvements observed in hematological and iron parameters were substantial, with hemoglobin rising by nearly 2.8 g/dL, TSAT increasing approximately twofold, and ferritin increasing more than tenfold. These changes mirror outcomes reported in other populations treated with iron isomaltoside, such as in the study by Kalra et al. (2020), where IV iron showed greater efficacy over oral iron [11]. The marked ferritin increase and TSAT normalization reflect effective replenishment of iron stores and support the role of iron isomaltoside 1000 as a rapid-acting IV iron therapy. This is particularly relevant in cancer patients, where anemia can impact QoL and chemotherapy tolerance. A systematic literature review and adjusted indirect treatment comparison (ITC) conducted by Pollock and Muduma (2019) revealed that iron isomaltoside resulted in a significantly larger increase in hemoglobin compared to ferric carboxymaltose, with a mean difference of +0.249 g/dL, though no significant difference was observed in the proportion of patients achieving a clinically relevant response. These findings further support the efficacy of iron isomaltoside in rapidly improving iron parameters in IDA patients [12]. Additionally, a prospective observational study by Pandya and Patel (2022) showed that the wide dosing range of iron isomaltoside 1000 allows for rapid iron correction in a single visit, providing both convenience and effectiveness for patients with IDA. This flexibility in dosing makes iron isomaltoside a particularly useful option in clinical settings where rapid restoration of iron stores is required, with minimal risk of adverse effects [13].

AEs in our study were infrequent (7%) and mostly involved transient Fishbane reactions, aligning with the known safety profile of iron isomaltoside. No serious hypersensitivity or anaphylactic reactions were observed in our study, which is consistent with previous reports by Paul et al. (2025) and Kalra et al. (2016), both of which documented a low incidence of AEs that were generally mild and non-severe [10,14]. Our results further confirm the high tolerability of iron isomaltoside 1000 in oncology settings. Given the immunocompromised nature of this population, the absence of major infusion-related complications reinforces the safety of single high-dose regimens.

Iron isomaltoside 1000 demonstrates potential as a safe, efficient, and logistically simplified option for IDA correction in cancer care. The retrospective multicenter design, modest sample size, lack of a comparator group, incomplete paired laboratory follow-up, and reliance on routine medical records may limit the interpretation and generalizability of the findings. In addition, AE severity grading, timing, and management details were not uniformly documented, and the exact dosing calculation method was not consistently captured as a fully standardized formula in the source records. Some supportive literature cited for context derives from non-oncology populations; therefore, cross-population comparisons should be interpreted cautiously. Future studies should include prospective multicenter cohorts, comparator arms, longer follow-up, and subgroup analyses by cancer type and treatment phase to better define optimal dosing strategies and long-term outcomes.

## Conclusions

IV iron isomaltoside 1000 demonstrated significant improvement in hematologic and iron parameters with a favorable safety profile in cancer patients with IDA. These findings suggest that iron isomaltoside 1000 may be effective and safe in correcting IDA in oncology patients in real-world settings; however, larger prospective studies are warranted to further confirm these results.
